# Expression characteristics of dual-promoter lentiviral vectors targeting retinal photoreceptors and Müller cells

**Published:** 2010-05-27

**Authors:** Susan L. Semple-Rowland, William E. Coggin, Mero Geesey, Kristofer S. Eccles, Leah Abraham, Krunal Pachigar, Rachel Ludlow, Shahrokh C. Khani, W. Clay Smith

**Affiliations:** 1 Department of Neuroscience, University of Florida McKnight Brain Institute, Gainesville, FL; 2 Schepens Eye Research Institute, Boston, MA; 3 Department of Ophthalmology, University of Florida, Gainesville, FL

## Abstract

**Purpose:**

Growing evidence suggests that successful treatment of many inherited photoreceptor diseases will require multi-protein therapies that not only correct the genetic defects linked to these diseases but also slow or halt the related degenerative phenotypes. To be effective, it is likely that therapeutic protein expression will need to be targeted to specific cell types. The purpose of this study was to develop dual-promoter lentiviral vectors that target expression of two proteins to retinal cones and rods, rods only, or Müller cells.

**Methods:**

Dual-promoter lentivectors were constructed using the following promoters: *Xenopus* opsin promoter (XOPS)1.3, murine opsin promoter (MOPS), interphotoreceptor retinoid binding protein promoter (IRBP156), rhodopsin kinase (RK), neural retina leucine zipper (NRLL), vimentin (VIM), cluster differentiation (CD44), and glial fibrillary acidic protein (GFAP). Vectors were packaged and injected into the neural tubes of chicken embryos. The activities of the promoters alone, in duplicate, or when paired with a different promoter were analyzed in transduced, fully-developed retinas, using direct fluorescent and immunofluorescent microscopy.

**Results:**

IRBP156, NRLL, and RK were active in cones and rods while XOPS1.3 was active only in rods. Of the glial promoters, only GFAP activity was restricted to Müller cells; both VIM and CD44 were active in Müller and neural cells. Dual-promoter vectors carrying IRBP156 and RK or XOPS1.3 and MOPS, in the order listed, exhibited robust expression of both reporter transgenes in cones and rods or rods only, respectively. Expression of the upstream transgene was much lower than the downstream transgene in dual-promoter vectors constructed using two copies of either RK or IRBP156. Analyses of the expression of a dual-promoter vector carrying CD44 and VIM in the order listed showed that the activity of the VIM promoter was more restricted to glial cells when paired with the CD44 promoter, while the activity of the CD44 promoter was inhibited to the extent that no CD44-driven reporter protein was detected in transduced cells.

**Conclusions:**

We have identified two dual-promoter vectors, one that targets cones and rods and one that targets rods alone. Both vectors reliably express the two proteins encoded by the transgenes they carry. When two well matched promoters are not available, we found that it is possible to target expression of two proteins to single cells using dual-promoter vectors carrying two copies of the same promoter. These vectors should be useful in studies of retina when co-delivery of a reporter protein with an experimental protein is desired or when expression of two exogenous proteins in targeted cells is required.

## Introduction

Treatment strategies for inherited photoreceptor disease often involve delivery of normal copies of the diseased gene to the affected photoreceptor cells. This unimodal gene-based treatment approach has been shown to restore function to nonfunctioning photoreceptors [1-3] and sight to blind animals, but in many cases the benefits have been relatively short lived, slowing but not preventing the natural course of the disease [4]. An alternative strategy that merits further investigation is to identify complementary or synergistic therapies that, when combined with corrective gene therapies, yield greater and more prolonged therapeutic or even curative benefits. Neurotrophic and anti-apoptotic therapies fit nicely into multimodal photoreceptor treatment strategies since these agents, when administered alone [5-10] or in combination with corrective gene therapies [11-13], have been shown to slow many retinal diseases. The full power of these therapies is likely to be realized only when these combination treatments are specifically targeted to the cells requiring them.

Lentiviral vectors have been the vector of choice in many applications requiring expression of multiple proteins in single cells. In most of these applications, the goal has been to produce vectors that express comparable levels of all of the proteins encoded by the vector transgene, a goal that remains one of the most significant hurdles facing developers of polycistronic vectors. Several approaches have been used to obtain multiple proteins from a single vector including insertion of internal ribosome entry sites, or 2A “cleavage” peptide sequences between the cistrons [14,15], and construction of vectors that carry multiple independent transcriptional units [16,17]. All of these approaches have proven useful, but their successful implementation has often required extensive paradigm-specific optimization of the vectors.

We recently completed a study to investigate the feasibility of using dual-promoter lentiviral vectors to achieve targeted expression of two proteins from a single vector. In that study we found that pairing the murine interphotoreceptor retinoid binding protein promoter (IRBP1783) and the chicken guanylate cyclase activating protein 1 promoter (GCAP292) resulted in specific expression of both cistrons in cone cells. Pairing IRBP1783 with the murine opsin promoter (MOPS) resulted in expression of the IRBP1783 cistron in cone cells and expression of the MOPS cistron in rod cells [18]. The goal of the current study was to determine if we could develop dual-promoter lentiviral vectors that specifically target cones and rods, rods only, or Müller cells. Several vectors were developed using four additional photoreceptor promoters and three putative glial promoters in chicken retina. The promoters exhibiting the desired activity profiles were then paired and used to construct several dual-promoter vectors whose activity profiles were also examined in vivo. Our efforts resulted in construction of two vectors, one that specifically targets cones and rods and one that targets rods only, both of which should be useful in situations requiring expression of two exogenous proteins in these cells.

## Methods

### Animals

All animal protocols were approved by the University of Florida Institutional Animal Care and Use Committee and adhered to the policies outlined in the Guide for the Care and Use of Laboratory Animals [19]. All eggs used in this study were obtained from our wild-type Rhode Island Red and our GUCY1*B breeding colonies that we maintain at the University of Florida. The GUCY1*B chickens carry a null mutation in the guanylate cyclase–1 gene [4,20].

### Embryonic injections

All lentiviral vectors were injected into embryonic day 2 (E2) chicken embryos (stage 9–11 [21]) as previously described [18]. Briefly, approximately 345 nl of virus was injected into the anterior neural tube. In instances where two different viruses were co-injected, equal volumes of virus were mixed together and were then injected. Following injection, eggs were sealed with Parafilm M (Pechinery Plastic Packaging, Chicago, IL) and incubated to E20. For these experiments 10–15 eggs were injected with each virus preparation. Approximately 20%–50% of the injected embryos survived to E20, and of those the retinas of approximately 50%–80% exhibited sufficient levels of viral transduction to permit analyses of the expression characteristics of the virus. A minimum of two treated embryos (four retinas) were analyzed for each vector at E20, an age when the chicken retina is fully-developed.

### Construction of lentiviral vectors

All lentiviral transducing vectors were designed using SimVector 4.01 (Premier Biosoft International; Palo Alto, CA). Fragments used in constructing vectors were obtained using either standard restriction enzyme digests or were amplified from existing vectors using primers shown in Table 1 and Pfu DNA polymerase (Stratagene; La Jolla, CA). Vector backbones used in non-directional ligations were dephosphorylated using shrimp alkaline phosphatase (Promega; Madison, WI) before ligation. Ligations were performed using a Quick Ligation kit (New England BioLabs; Ipswich, MA). The desired vectors were identified by restriction analyses of plasmid DNA isolated from One Shot TOP10 chemically competent *Escherichia coli* cells (Invitrogen, Carlsbad, CA) that had been transformed with the ligation mixtures. The maps and sequences of all of the vectors described in this study are available online through everyVECTOR. Shared access to this information can be obtained by contacting the corresponding author.

**Table 1:** PCR primers for lentivirus vector constructs

**Vector insert**	**Primer name**	**Sequence**
RK		
	S-BamHI	TTAGGATCCGGGCCCCAGAAGCCT
	S-EcoRV	TTAGATATCGGGCCCCAGAAGCCT
	AS- KpnI	ATTGGTACCGCCCTTGGCCTGTGG
	AS-PmeI	ATTGTTTAAACGCCCTTGGCCTGTGG
XOPS		
	S-NheI	ATTGCGGCCGCAGATCTTTATACATTGCT
	AS-NheI	AATGCTAGCCTCGAGATCCCTAGAAGC
NRLL		
	S-NotI	TTGCGGCCGCGCGCTACCGGACTCA
	AS-NheI	GCGCTAGCGGCGACCGGTGGAT
EFIα		
	S-NotI	ATTGCGGCCGCTTTGGAGCTAA
	AS-NotI	TTAGCGGCCGCCACGACACCTGAAAT
WPRE		
	S-SpeI	ATAACTAGTATAATCAACCTCTGGATT
	S-MluI	ATAACGCGTATAATCAACCTCTGGATT
	S-KpnI	GGTGGTACCATAATCAACCTCTGGATT
	AS-SpeI	ATAACTAGTGGTCGACGGTATCGATGC
	AS-MluI	ATAACGCGTGGTCGACGGTATCGATGC
	AS-KpnI	ATAGGTACCGGTCGACGGTATCGATGC
mCherry		
	S-NheI	AAAGCTAGCAAGGATCCCGCCACC
	AS-NheI,MluI	AAAGCTAGCACGCGTCGTACGTCGGGCTTTGT
GFP		
	S-NheI	GCAGCTAGCCGCCACCATGAGCAA
	AS-NheI, MluI	AATGCTAGCACGCGTCGTACGAGAGGCCTCAG
	S-XhoI	CCTCTCGAGCGCCACCATGAGCAA
	AS-XhoI	AATCTCGAGAGAGGCCTCAGTCAG
IRBPI56		
	S-NheI	TTGCGGCCGCGCGCTACCGGACTCA
	AS-NotI	GCGCTAGCGGCGACCGGTGGAT
RK-GFP		
	S-SpeI	ATACTAGTGGGCCCCAGAAGCCT
	AS-SpeI, MluI	
	S-XhoI	ATCTCGAGGGGCCCCAGAAGCCT
	AS-XhoI	ATCTCGAGGATATCTTACTTGTACAGCTCGTCC
tdTomato		
	S-PmeI	AATGATGTTTAAACCTGTACGACGATGACGATAAG
	AS-SalI	TTACATGTCGACTCCTTTCGGGCTTTGTTA
Vimentin		
	S-MluI	GGCACGCGTCTGTTTTACCCACCATCTCAGTTCTAATATT
	AS-MluI	GGCACGCGTGGTACCCGGGGATCCACTAGTTCTAGAAAT

### Vector elements

The pFIN lentiviral vector backbone used in this study that includes a woodchuck hepatitis post-transcriptional regulatory element (WPRE), two copies of the 250 bp core region of chicken β-globin insulator (HS4), and a central polypurine tract and central termination sequence (cPPT/CTS) that creates a plus strand overlap of the vector DNA (FLAP) during reverse transcription of the viral RNA, has previously been described [18]. The following promoters were used in our constructs: cluster differentiation (CD) 44, elongation factor-1α (EF1α), glial fibrillary acidic protein (GFAP), guanylate cyclase activating protein-1 (GCAP) 292, interphotoreceptor retinoid binding protein promoter (IRBP) 156 and 1783, murine opsin promoter (MOPS), neural retina leucine zipper (NRLL), rhodopsin kinase (RK), vimentin (VIM) and the *Xenopus* opsin promoter (XOPS)1.3. We used the fluorescent reporter proteins tdTomato (tdTOM), mCherry (CHER), and green fluorescent protein (GFP) to monitor the activities of the promoters in chicken retina.

### Photoreceptor promoter vectors

pFIN-IRBP1783-tdTOM: This vector was constructed as previously described [18].

pFIN-IRBP156-tdTOM: tdTOM was excised from pFIN-IRBP1783-tdTOM using PmeI and SalI sites and was ligated into the pFIN backbone [18]. pFIN-tdTOM was then cut with NotI and NheI and the mouse IRBP156 (350 bp) promoter, which was amplified using sense and antisense primers containing NotI and NheI sites, respectively, and the IRBP1783 promoter as a template, was ligated into pFIN-tdTOM to create pFIN-IRBP156-tdTOM.

pFIN-RK-GFP-WPRE: The human rhodopsin kinase (RK) promoter extending from −112 to +180 (292 bp) [22] was amplified from p150E1E2EG using sense and antisense primers containing BamHI and KpnI sites, respectively. The RK promoter was ligated into pFIN that had been digested with BamHI and KpnI. The resulting pFIN-RK vector was cut with KpnI. GFP-WPRE (GW) was removed from pFmCD44.1GW (kindly provided by John Flannery, University of California, Berkeley, CA) using KpnI and was ligated into pFIN-RK to create pFIN-RK-GFP-WPRE.

pFIN-XOPS1.3-tdTOM: pFIN-EF1α-tdTOM, an intermediate vector generated during construction of EF1α-tdTOM-GCAP292-GFP [18], was digested with NotI and NheI to remove the EF1α promoter. The XOPS1.3 promoter (1370 bp) [23] was amplified from XOPS1.3p44/GFP using sense and antisense primers containing NotI and NheI sites, respectively, digested with NotI and NheI, and ligated into pFIN-tdTOM to create pFIN-XOPS1.3-tdTOM.

pFIN-NRLL-tdTOM: pFIN-EF1α-tdTOM was digested with NotI and NheI to remove EF1α. The mouse neural retina leucine zipper (NRLL) promoter (2,591 bp) [24] was amplified from pEGFP1-pNRLL (kindly provided by Anand Swaroop, National Eye Institute, Bethesda, MD) using sense and antisense primers containing NotI and NheI sites, respectively, and was ligated into pFIN-tdTOM to create pFIN-NRLL-tdTOM.

pFIN-GCAP292-GFP: GCAP292-GFP was obtained from pFIN-EF1α-tdTOM-GCAP292-GFP [18] using SalI and was ligated into our pFIN transducing vector that had been linearized with SalI.

### Non-photoreceptor promoter vectors

pFIN-CD44-GFP-WPRE: pFmCD44.1GW [25] (kindly provided by John Flannery, University of California, Berkeley, CA) was digested with BamHI and KpnI to remove the murine CD44 promoter (BamHI fragment) and GFP-WPRE (KpnI fragment). Our pFIN transducing vector was digested with BamHI, and CD44 was ligated into the vector. The resulting pFIN-CD44 vector was then digested with KpnI, and GFP-WPRE was ligated into it to create pFIN-CD44-GFP-WPRE.

pFmVIMGW and pFmGFAPGW: The pFmVIMGW and pFmGFAPGW lentiviral vectors [25] containing the murine vimentin (VIM) and GFAP promoters driving enhanced GFP (G), respectively, followed by a WPRE element (W), were kindly provided by John Flannery (University of California, Berkeley, CA).

pFIN-EF1α-CHER-WPRE: EF1α (1,458 bp) was amplified from pFIN-EF1α-tdTOM using sense and antisense primers containing NotI sites and was ligated into pFIN-WPRE that was linearized with NotI. mCherry (CHER: 790 bp) was amplified from pRSET-B-mCherry (kindly provided by Roger Tsien, University of California, San Diego) using sense and antisense primers containing NheI and NheI and MluI, respectively. CHER was then digested with NheI and ligated into pFIN-EF1α-WPRE that had been linearized with NheI.

pFIN-EF1α-GFP-WPRE: pFIN-EF1α-GFP was digested with NheI to remove GFP. A unique MluI site was added to the vector by re-amplifying GFP (735 bp) using sense and antisense primers containing NheI, and NheI and MluI sites, respectively, digesting the amplified GFP with NheI, and ligating it back into pFIN-EF1α. WPRE (625 bp) was amplified from pFIN-RK-GFP-WPRE using sense and antisense primers containing MluI sites. Finally, pFIN-EF1α-GFP and the WPRE were digested with MluI and ligated together to create pFIN-EF1α-GFP-WPRE.

### Dual promoter vectors

All dual promoter vectors were constructed by inserting two transgenes, each consisting of a promoter plus a reporter, into the multiple cloning site of our pFIN transducing vector backbone. The transgenes were arranged head-to-tail so that transcription of both transgenes proceeded in the same direction. The transgenes shared the same polyadenylation site, which was located in the dl.R region of the 3′ long-terminal repeat [18].

pFIN-RK-GFP-IRBP156-CHER-WPRE: CHER was removed from pRSET-B-mCherry using BamHI and EcoRI and was ligated into our pFIN transducing vector. The resulting pFIN-CHER vector was digested with NotI and NheI. IRBP156 (350 bp) was amplified from pFIN-IRBP1783 using a sense and antisense primers containing NotI and NheI sites, respectively, and was ligated into the pFIN-CHER vector to create pFIN-IRBP156-CHER. RK-GFP (1,040 bp) was amplified from pFIN-RK-GFP-WPRE using sense and antisense primers containing XhoI sites. pFIN-IRBP156-CHER and RK-GFP were digested with XhoI and were ligated together. WPRE (613 bp) was amplified from pFmCD44.1GW using sense and antisense primers containing SpeI sites. pFIN-RK-GFP-IRBP156-CHER and WPRE were digested with SpeI and ligated together to create the final vector.

pFIN-IRBP156-CHER-RK-GFP-WPRE: RK-GFP (1,040 bp) was amplified from pFIN-RK-GFP-WPRE using sense and antisense primers containing SpeI and SpeI and MluI sites, respectively. RK-GFP and pFIN-IRBP156-CHER, an intermediate vector in the construction of pFIN-RK-GFP-IRBP156-CHER-WPRE, were digested with SpeI and ligated together to create pFIN-IRBP156-CHER-RK-GFP. Finally, WPRE flanked with MluI sites (described above) was ligated into pFIN-IRBP156-CHER-RK-GFP that had been digested with MluI.

pFIN-RK-GFP-RK-CHER-WPRE: pFIN-CHER was digested with XhoI. RK-GFP was removed from pCR2.1-RK-GFP using XhoI and ligated into pFIN-CHER, creating pFIN-RK-GFP-CHER. A second copy of the RK (312 bp) promoter was amplified from pFIN-RK-GFP-WPRE using sense and antisense primers containing EcoRV and PmeI sites, respectively. pFIN-RK-GFP-CHER and the RK promoter were digested with EcoRV and PmeI and were ligated together to create pFIN-RK-GFP-RK-CHER. Finally, WPRE was amplified with primers containing SpeI sites and was ligated pFIN-RK-GFP-RK-CHER that had been linearized with SpeI.

pFIN-IRBP156-CHER-IRBP156-GFP-WPRE: pFIN-IRBP156-CHER was digested with SpeI. IRBP156-GFP (1,119 bp) was amplified from pFIN-IRBP1783-GFP using a sense primer containing SpeI and EcoRV sites and an antisense primer containing SpeI and MluI sites.The amplified IRBP156-GFP was digested with SpeI and ligated into pFIN-IRBP156-CHER to create pFIN-IRBP156-CHER-IRBP156-GFP. Finally, WPRE, amplified from pFIN-RK-GFP-WPRE using sense and antisense primers containing MluI sites, and pFIN-IRBP156-CHER-IRBP156-GFP were digested with MluI and ligated together to create the final vector.

pFIN-XOPS-tdTOM-MOPS-GFP: pFIN-XOPS-tdTOM was linearized with SalI. MOPS-GFP was extracted from pFIN-MOPS-GFP [18] using SalI sites and was ligated into pFIN-XOPS-tdTOM to form pFIN-XOPS-tdTOM-MOPS-GFP.

pFIN-MOPS-GFP-XOPS-tdTOM-WPRE: pFIN-XOPS-tdTOM was digested with KpnI. WPRE was amplified from pFIN-WPRE (628 bp) using sense and antisense primers containing KpnI sites and was ligated into pFIN-XOPS-tdTOM. MOPS was excised from pFIN-MOPS-GFP [18] using NotI and ligated into pFIN-XOPS-tdTOM that had been linearized with NotI to create pFIN-MOPS-XOPS-tdTOM-WPRE. Finally, GFP (763 bp) was amplified from pFIN- EF1α-GFP using sense and antisense primers containing XhoI sites and ligated into pFIN-MOPS-XOPS-tdTOM-WPRE that had been linearized using XhoI to create pFIN-MOPS-GFP-XOPS-tdTOM-WPRE.

pFIN-CD44-CHER-VIM-GFP-WPRE: pFIN-CD44-GFP-WPRE was linearized using AgeI. CHER (797 bp) was amplified from pFIN-IRBP1783-CHER using sense and antisense primers containing AgeI and AgeI, and MluI sites, respectively, and ligated into pFIN-CD44-GFP-WPRE to create pFIN-CD44-CHER-GFP-WPRE. The VIM promoter (3,195 bp) was amplified from pFmVIMGW using sense and antisense primers containing MluI sites and ligated into pFIN-CD44-CHER-GFP-WPRE that had been linearized with MluI to form pFIN-CD44-CHER-VIM-GFP-WPRE.

### HS4 vectors

pFIN-RK-GFP-HS4(2×250)F-IRBP156-CHER-WPRE: pFIN-RK-GFP-IRBP156-CHER was digested with EcoRV. pNI-CD (kindly provided by Gary Felsenfeld, National Institutes of Health, Bethesda, MD) was cut with KpnI to obtain the 500 bp HS4(2×250) insulator. HS4(2×250) was treated with T4 DNA polymerase and ligated into pFIN-RK-GFP-IRBP156-CHER in the forward (F) orientation. WPRE (613 bp) was amplified from pFmCD44.1GW using sense and antisense primers containing SpeI sites and was ligated into the pFIN-RK-GFP-HS4(2×250)F-IRBP156-CHER vector that had been linearized using SpeI.

pFIN-RK-GFP-HS4(1.2)F-IRBP156-CHER-WPRE: pNI-CD was cut with XbaI to obtain the 1.2-kb HS4 fragment. The construction of this vector was the same as that used for pFIN-RK-GFP-HS4(2×250)F-IRBP156-CHER except that the HS4 1.2-kb fragment was ligated into pFIN-RK-GFP-IRBP156-CHER in the forward orientation. WPRE (613 bp) was amplified from pFmCD44.1GW using sense and antisense primers containing SpeI sites and was ligated into the pFIN-RK-GFP-HS4(1.2)F-IRBP156-CHER vector that had been linearized using SpeI.

### Packaging and titering lentiviruses

The lentiviral vectors described above were packaged into vesicular stomatitis virus G (VSV-G) glycoprotein pseudotyped lentivirus, using a three plasmid packaging system as previously described [18,26]. The titer of each lentivirus (viral genomes/µl) was determined using a Lenti-X qRT–PCR titration kit (Clontech, Mountain View, CA). The average titer of the viruses used in this study was 7.45×10^12^ genomes/ml ranging from 1.85×10^10^ to 5.6×10^13^.

### Retinal whole-mounts and immunohistochemistry

Retinal whole mounts were prepared as previously described [18]. The GFP reporter protein was detected by examining the whole mounts with a narrow-band GFP filter set 41020 (Chroma Technology Corp, Bellows Falls, VT); CHER and tdTOM reporter proteins were detected using a Chroma custom filter set that consisted of an exciter ET572/35, an emitter ET632/60, and a beamsplitter (Chroma Technology Corp, Bellows Falls, VT). The expression patterns of the reporter proteins in the retinal whole mounts were documented using a Zeiss AxioCam MRm digital camera (Carl Zeiss Microimaging, Inc.,Thornwood, NY). In selecting the representative images shown in the figures, our goal was to document all of the cell types and the variability in expression levels of the reporter proteins observed in transduced cell populations. Co-expression of the reporter proteins was analyzed using the co-localization module of the Zeiss AxioVision Image analysis suite. The results of these analyses were plotted using GraphPad Prism 5.02 (GraphPad Software, Inc., La Jolla, CA).

For immunohistochemistry, the retinas were equilibrated in 30% sucrose in phosphate buffered saline (137 mM NaCl, 2.7 mM KCl, 100 mM Na_2_HPO_4_, 2.0 mM KH_2_PO_4_) overnight, cryosectioned at 10 µm, and either stained with 4’,6-diamidino-2-phenylindole (DAPI) alone or in combination with a rod transducin polyclonal antibody (1:1,000; G-t1: K20; Santa Cruz Biotechnology, Inc., Santa Cruz, CA) or a mouse monoclonal antibody against chicken carbonic anhydrase II (CAII; 1:500: kindly provided by Paul Linser, University of Florida, Gainesville, FL). The primary antibodies were localized using either Alexa Fluor 488 or 594 secondary antibodies (Invitrogen). Zeiss filter set 02 was used to visualize DAPI-stained cell nuclei. In experiments in which virus expression was examined in brain, 10 µm sagittal brain sections were stained with a chicken anti-GFP antibody (1:1,000; ab13970; Abcam, Cambridge, MA) to enhance visualization of the GFP expressing cells and DAPI.

## Results

**Figure 1:**
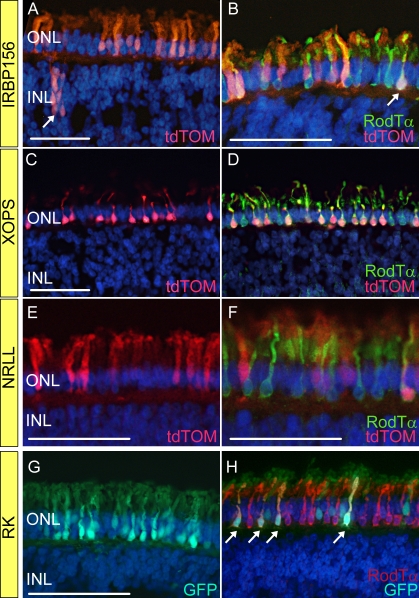
Cellular activities of interphotoreceptor retinoid binding protein promoter (IRBP156), *Xenopus* opsin promoter (XOPS), neural retina leucine zipper (NRLL), and rhodopsin kinase (RK) promoters in chicken retina. Lentiviral vectors were injected into the ventricles of chicken embryos (embryonic day 2 –E2) in ovo. The viruses injected were as follows: **A-B** pFIN-IRBP156-tdTOM; **C-D** pFIN-XOPS-tdTOM; **E**-**F** pFIN-NRLL-tdTOM; **G-H** pFIN-RK-GFP-WPRE. The retinas of the injected embryos were harvested on E19–20 and the cells expressing the fluorescent reporter proteins were identified using native fluorescent and immunofluorescent microscopy. In selecting the representative images shown in this and subsequent figures, our goal was to document all of the cell types and the variability in expression levels of the reporter proteins observed in transduced cell populations. The sections shown in **B**, **D**, **F,** and **H** were immunostained with a rod transducin polyclonal antibody that was visualized using either goat anti-rabbit Alexa Fluor 488 (**B**, **D**, **F**) or 594 (**H**) secondary antibody. Arrows indicate inner retinal cells (**A**) or rod photoreceptors (**B**, **H**). All sections were counterstained with DAPI. All scale bars shown equal 50 µm. Abbreviations: ONL represents outer nuclear layer; INL represents inner nuclear layer.

To develop dual-promoter vectors that specifically target both cones and rods or that target rods only, we first needed to identify promoters that exhibited these expression characteristics in vivo. To accomplish this, we constructed four lentiviral vectors, each carrying a fluorescent reporter protein whose expression was driven by IRBP156, XOPS, NRLL, or RK (Figure 1). The first promoter examined was mouse IRBP156, a truncated version of IRBP1783 whose activity has been shown to be restricted to photoreceptors [27,28], which we found to exhibit cone-specific activity in chicken retina [18]. In retinas transduced with pFIN-IRBP156-tdTOM lentivirus, tdTOM was detected in the photoreceptor cells and occasionally in groups of cells in the inner nuclear layer (Figure 1A). Sections of these retinas counterstained with an antibody against rod transducin alpha revealed that unlike IRBP1783, IRBP156 was active in both rod and cone cells (Figure 1B). The second promoter examined was the *Xenopus* opsin promoter, XOPS, a promoter that exhibits rod-specific activity in frog retina [23]. In retinas transduced with pFIN-XOPS1.3-tdTOM, tdTOM expression was restricted to rod cells, as evidenced by tdTOM and rod transducin alpha co-localization (Figure 1C,D). This expression pattern was identical to that which we previously observed for the mouse opsin promoter MOPS [18]. The third promoter examined was mouse NRLL, the activity of which is restricted to rods in mouse retina [24]. In fully differentiated chicken retinas transduced with pFIN-NRLL-tdTOM, expression of tdTOM was restricted to the photoreceptor layer but was not limited to rod cells. Unlike its activity profile in mouse retina, NRLL exhibited activity in both cones and rods in chicken retina (Figure 1E,F). The expression pattern of the mouse NRLL promoter in chicken cones and rods was unexpected. Searches performed by our laboratory and others indicate that there are no well conserved homologs of NRL in the chicken genome [29,30]. However, NRL, which is a member of the Maf-family of transcription factors, does exhibit extended homology with the chicken v-maf musculoaponeurotic fibrosarcoma oncogene homolog K (MAFK) that encodes v-maf [31]. Thus, the activity of the mouse NRLL promoter in chicken cones and rods may reflect differences in the transactivation of this promoter in birds and mammals. Finally, the fourth promoter examined was human RK, a promoter that is active in both cones and rods in mouse and rat retinas [22,32]. In chicken retinas transduced with pFIN-RK-GFP-WPRE, GFP expression was detected primarily in cones and rods (Figure 1G,H) and was occasionally observed in scattered cells within the inner nuclear layer. In sum, of the four photoreceptor promoters examined in this series of experiments, IRBP156, NRLL, and RK exhibited activity in both cones and rods while XOPS activity was restricted to rods (see Table 2 for summary).

**Table 2:** Summary of promoter activities in chicken retina

** **	**Cells exhibiting promoter activity**					
**Promoters**	**Ganglion cells**	**Müller cells**	**Inner nuclear layer**	**Photoreceptors**	**Rods**	**Cones**
IRBP156			x		x	x
XOPS					x	
NRLL					x	x
RK					x	x
CD44	x	x	x	x		
VIM		x	x	x		
GFAP		x				

**Figure 2:**
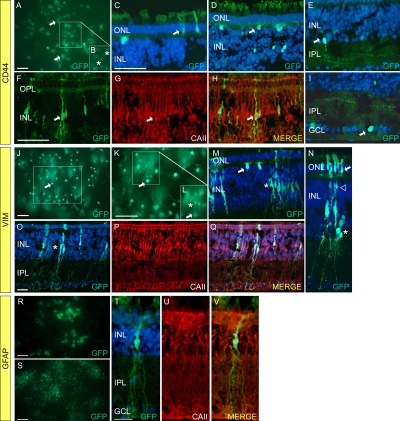
Cellular activities of cluster differentiation (CD)44, vimentin (VIM), and glial fibrillary acidic protein (GFAP) promoters in chicken retina. Lentiviral vectors were injected into the ventricles of chicken embryos (embryonic day 2–E2) in ovo. The retinas of the injected embryos were harvested on E19–20 and the cells expressing the fluorescent reporter proteins were identified using native fluorescent and immunofluorescent microscopy. The viruses injected were as follows: **A-I** pFIN-CD44-GFP-WPRE; **J-Q** pFM-VIM-GW; **R-V** pFM-GFAP-GW. All sections were counterstained with DAPI, and all scale bars shown equal 50 µm. Abbreviations are as follows: ONL represents outer nuclear layer; INL represents inner nuclear layer; IPL represents inner plexiform layer; GCL represents ganglion cell layer. CD44: **A**: Photograph of whole mount of retina that had been treated with pFIN-CD44-GFP-WPRE. Clusters of GFP-positive photoreceptors (arrows) were detected across the surface of the whole mount. **B**: This image was produced by re-photographing the boxed region shown in A using a focal plane just below that used to obtain the image shown in A. Horizontal cells (asterisks) were the predominant GFP-positive cell type observed in this focal plane. **C-I** These images represent sections of retinas showing the cell types (arrows) in which the CD44-GFP transgene was active (**C** photoreceptors, **D** horizontal cells, **E** amacrine cells, **F-H** Müller cells, **I** ganglion cells). Section shown in **F** was counterstained with an antibody against chicken carbonic anhydrase II (CAII), a marker for Müller cells (**G**). The merged image (**H**) shows that the GFP-positive cells also expressed carbonic anhydrase II. VIM: **J**,** K** Photographs of a whole mount of a retina treated with pFM-VIM-GW and viewed from the photoreceptor side of the whole mount. **J** Numerous GFP-positive horizontal cells were detected in the transduced retina (arrow). **K** Enlargement of the region in image J (box) that contains GFP-positive horizontal cells (arrow). **L** This image was produced by re-photographing the boxed region shown in K using a focal plane just below that shown in **K**. Müller cell bodies are the predominant cell type observed in this image plane (asterisk). The horizontal cell indicated in **J**, **K,** and **L** by the arrow is the same cell. **M,N** Images of sections of the retinal whole mount shown in **J** and **K**. GFP-positive horizontal (**M**, arrow), Müller (**M**, asterisk), and photoreceptor (**N**, ONL) cells were detected in several sections. **O-Q** A section containing GFP-positive cells located in the INL (**O**, arrow) was counterstained with an antibody against chicken carbonic anhydrase II (**P**). The merged image (**Q**) shows that the GFP-positive cells also expressed carbonic anhydrase II. **GFAP: R**, **S** Images of a whole mount of a 5-week old GUCY1*B chicken retina that had been treated with pFM-GFAP-GW on E2 and photographed from either the photoreceptor (**R**) or the vitread (**S**) side of the whole mount. The pattern of GFP localization observed in these whole mounts suggested that the cells expressing the GFAP-GFP transgene were Müller cells. **T-V** Sections of the transduced retinas showed that the cell bodies of the GFP-positive cells observed in R and S were located in the INL (**T**). Immunostaining of these sections with an antibody against chicken carbonic anhydrase II (**U**) revealed that the GFP-positive cells also expressed carbonic anhydrase II (**V**).

In addition to photoreceptor targeted vectors, we also wanted to develop dual-promoter vectors that specifically target Müller cells. As a first step toward this goal, we analyzed the expression characteristics of the murine CD44, VIM, and GFAP promoters in chicken retina (Figure 2). All three of these promoters have been reported to drive expression of lentiviral transgenes in Müller cells in rat retina [25]. To examine the expression characteristics of CD44, we removed the promoter from pFmCD44.1GW [25] and inserted it into our lentiviral backbone to create pFIN-CD44-GFP-WPRE. In retinas transduced with lentivirus prepared using this vector, GFP expression was detected in numerous retinal cell types, including photoreceptors (Figure 2C), horizontal cells (Figure 2D), amacrine cells (Figure 2E), Müller cells (Figure 2F-H) and ganglion cells (Figure 2I). The cellular specificities of the VIM and GFAP promoters in chicken retina were determined by examining GFP expression in retinas transduced with lentiviruses prepared using pFmVIMGW or pFmGFAPGW lentivectors [25], respectively. VIM-driven GFP expression in retinas transduced with pFMVIMGW was primarily detected in horizontal cells (arrows; Figure 2J-M) and in Müller cells (asterisks; Figure 2L,M,O). GFP was also detected in photoreceptors (Figure 2N, arrow) and in cells in the inner nuclear layer, which were identified as bipolar cells (Figure 2N, open arrowhead) and amacrine cells (Figure 2N, asterisk), based on the position of their cell bodies within the inner nuclear layer [33]. In our initial experiment to examine the cellular specificity of the GFAP promoter, we found that levels of expression of the GFAP-GFP transgene in the retinas of E20 wild-type embryos that had been treated with pFmGFAPGW lentivirus was very low. This result is consistent with our previous study of GFAP expression in chicken retina in which we found that the activity of the GFAP promoter is low in normal retina and increases in the presence of injury or disease [34]. To better assess the expression characteristics of this promoter in chicken retina, we treated E2 GUCY1*B embryos with the pFmGFAPGW virus, hatched the chickens, and examined the retinas of the animals at 5 weeks, a time when retinal degeneration due to the absence of guanylate cyclase-1 is well underway [35]. Examination of the retinas of these animals revealed that GFP expression was restricted to Müller cells (Figure 2R-V). The results of these analyses, summarized in Table 2, show that the GFAP promoter exhibits the highest specificity for Müller cells in chicken retina.

We have recently shown that it is possible to deliver multi-protein therapies to retina using dual-promoter lentivectors carrying multiple transgenes [18]. Another approach that has been used to deliver multiple therapies to retina has been to administer a mixture of two or more viruses to the retina, each virus carrying a transgene whose expression is regulated by a promoter with well defined expression characteristics [11-13]. Before moving on to developing cell-specific dual-promoter vectors to deliver two therapeutic proteins to single cells, we wanted to determine if we could achieve this goal using mixtures of lentiviruses, each encoding a specific protein. To address this question, we injected mixtures of two lentiviruses into the neural tubes of E2 embryos and monitored expression of the reporter genes carried by the viruses in retina and brain.

**Figure 3:**
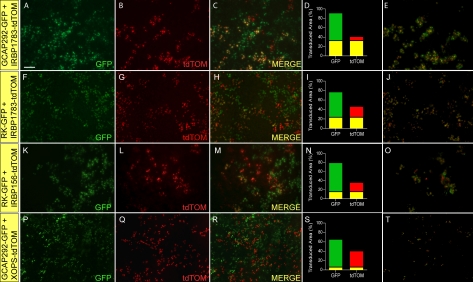
Expression of photoreceptor promoter-driven fluorescent proteins in retinas transduced with mixtures of two lentiviruses. Lentiviral vectors carrying transgenes comprised of various photoreceptor promoters driving expression of GFP or tdTOM fluorescent proteins were mixed in equal volumes and injected into the developing neural tubes of chicken embryos (embryonic day 2 –E2) in ovo. The injected virus mixtures were as follows: **A-E**: pFIN-GCAP292-GFP (2.2×10^10^ vector genomes/µl) and pFIN-IRBP1783-tdTOM (1.6×10^10^ vector genomes/µl); **F-J**: pFIN-RK-GFP-WPRE (1.2×10^10^ vector genomes/µl) and pFIN-IRBP1783-tdTOM (1.6×10^10^ vector genomes/µl); **K-O**: pFIN-RK-GFP-WPRE (1.2×10^10^ vector genomes/µl) and pFIN-IRBP156-tdTOM (5.6×10^10^ vector genomes/µl); **P-T**: pFIN-GCAP292-GFP (2.2×10^10^ vector genomes/µl) and pFIN-XOPS-tdTOM (1.2×10^9^ vector genomes/µl). We have previously shown that the GCAP292 and IRBP1783 promoters are active in cone cells [18]. RK and IRBP156 are active in both rod and cone cells and XOPS is only active in rod cells (Figure 1). For each image series, the transduced retina was photographed from the photoreceptor side of the whole mount using GFP (**A**, **F**, **K**, **P**) and CHER (**B**, **G**, **L**, **Q**) filters. These images were then merged to identify cells expressing both reporter proteins (**C**, **H**, **M**, **R**). The merged images were analyzed using the co-localization module of the Zeiss AxioVision Image Suite. The results of these analyses are expressed as the percent of the transduced area in the image (pixels) containing co-localized GFP and tdTOM (yellow bar) or GFP (green bar) or tdTOM (red bar) fluorescence alone (**D**, **I**, **N**, **S**). The images shown in **E**, **J**, **O**, and **T** were extracted from the merged images shown in **C**, **H**, **M**, and **R** and show only those areas of the merged image in which GFP was co-localized with tdTOM. The scale bar shown in **A** is applicable to all images and equals 50 µm.

In our first series of experiments, we paired lentiviruses carrying transgenes encoding fluorescent reporter proteins, the expression of which was driven by two different photoreceptor promoters possessing similar cellular activity profiles: (1) pFIN-GCAP292-GFP and pFIN-IRBP1783-tdTOM, (2) pFIN-RK-GFP-WPRE and pFIN-IRBP1783-tdTOM, and (3) pFIN-RK-GFP-WPRE and pFIN-IRBP156-tdTOM. We previously found that the GCAP292 and IRBP1783 promoters are active in cone cells [18] and in this study we found that RK and IRBP156 are active in both rod and cone cells (Figure 1A,B,G,H). Examination of retinal whole mounts treated with these virus mixtures revealed that both viruses effectively transduced the target tissues and that the expression of the fluorescent reporter proteins encoded by the transgenes carried by the viruses were easily detected in retinal whole mounts (Figure 3A-O). Within infected areas, GFP or tdTOM were observed in more than half of the cells, with little evidence of co-expression, a pattern suggesting that few cells had been infected by both viruses. The retinal areas shown in Figure 3 were selected because they contained transduced cells expressing both GFP and tdTOM. The percentages of the infected cells expressing both reporter proteins in these transduced areas were 33% (Figure 3D,E), 24% (Figure 3I,J), and 16% (Figure 3N,O). In addition to pairing lentiviruses carrying promoters with similar cellular activity profiles, we also paired lentiviruses carrying promoters with different cellular activity profiles: pFIN-GCAP292-GFP and pFIN-XOPS-tdTOM. GCAP292, as indicated above, is active in cones, while XOPS is specifically expressed in rod cells (Figure 1C,D). Our expectation was that very few, if any, cells would co-express GFP and tdTOM in retinas infected with this virus mixture. Analyses of these retinas showed that only 6% of the infected regions exhibited co-localization (Figure 3R,S), a result consistent with the expression profiles of these promoters.

**Figure 4:**
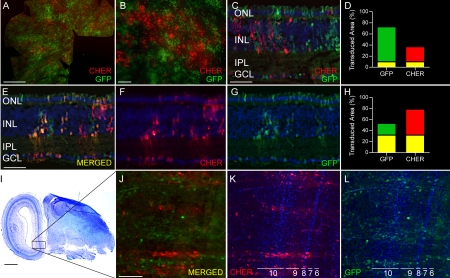
Expression of elongation factor 1a (EF1a) promoter-driven fluorescent proteins in retina and brain transduced with a mixture of two lentiviruses. Equal volumes of two lentiviral vectors, pFIN-EF1a-GFP-WPRE (2.4×10^10^ vector genomes/µl) and pFIN-EF1a-CHER-WPRE (4.6×10^9^ vector genomes/µl), were mixed and injected into the developing neural tubes of E2 chicken embryos in ovo. The retinas and brains of the injected embryos were harvested on E19-E20 and the cells expressing GFP and CHER were identified using fluorescent microscopy. **A**,** B** These retinal whole mounts, viewed from the photoreceptor side, show the distribution of retinal cells expressing GFP and/or CHER fluorescent protein(s). The scale bars in **A** and **B** equal 2000 and 50 µm, respectively. **C-D** and **E-H**: Sections of whole mounts shown in A and B show that the EF1a promoter is active in cells distributed throughout the neural retina. **C**: This retinal section, which contains very few transduced cells expressing both GFP and CHER, was typical of most regions of the transduced retinas. **D**: The image shown in C was analyzed using the co-localization module of the Zeiss AxioVision Image Suite. The results of these analyses are expressed as the percent of the transduced area in the image (pixels) containing co-localized GFP and CHER (yellow bar) or GFP (green bar) or CHER (red bar) fluorescence alone. **E-G**: Image of retinal section showing green (**E**), red (**F**) and merged (**G**) channels that contain several cells expressing both GFP and CHER. **H**: The image shown in **E** was analyzed using the co-localization module of the Zeiss AxioVision Image Suite. The results of this analysis are expressed as the percent of the transduced area in the image (pixels) containing co-localized GFP and CHER (yellow bar) or GFP (green bar) or CHER (red bar) fluorescence alone. The scale bars in C and D equal 50 µm. **I**: Thionin stained sagittal section of E20 chicken brain. Scale bar equals 1000 µm. **J-L**: Fluorescent images of GFP and CHER expression in optic tectum (the region shown in F-F’’ corresponds to the boxed region in **I**). The brain sections were stained with a chicken anti-GFP antibody to enhance visualization of the GFP expressing cells. Tectal layers are numbered according to Cajal [43]. Scale bar in **J** equals 100 µm. Retinal and brain sections shown in **C**, **D**-**G** and **J**-**L** were counterstained with DAPI.

In our second series of experiments, we asked if delivery of a mixture of two viruses carrying reporter proteins driven by identical ubiquitously expressed promoters would increase the number of co-infected cells, as evidenced by co-localization of the two reporter proteins. To address this question, we injected a mixture of pFIN-EF1α-CHER-WPRE and pFIN-EF1α-GFP-WPRE that encoded either CHER or GFP driven by the ubiquitous EF1α promoter and examined expression of the fluorescent proteins in retina and brain. Examination of treated retinal whole mounts revealed a patchwork of expression of GFP and CHER with little evidence of co-localization of these proteins (Figure 4A,B). Sections of these retinas revealed that GFP and CHER were distributed throughout all of the layers of the retina (Figure 4C,E-G). Most retinal sections showed little evidence of co-localization of GFP and CHER (Figure 4C,D). Analyses of the image in Figure 4C showed that approximately 10% of the total area expressing reporter protein (34% of the image) contained cells expressing both proteins (Figure 4D). In regions containing co-transduced cells, these cells were often clustered (Figure 4E-G), a distribution pattern consistent with viral infection of retinal progenitor cells in E2 embryos that would lead to expression of the viral transgenes in their daughter cells. Analyses of these regions showed that approximately 33% of the total area expressing reporter protein (30% of the image) contained cells expressing both proteins (Figure 4H). Because the virus pair used in this experiment carried the ubiquitously expressed EF1α promoter, we were able to examine transgene expression in the brains as well as the retinas of treated embryos. The expression patterns of GFP and CHER in brain were similar to those observed in retina. In highly organized brain regions, such as the optic tectum (Figure 4I box), both reporter proteins were detected, but among the columns of cells expressing these proteins, very few cell groups (3% of transduced area) were identified that co-expressed these proteins (Figure 4J-L). In sum, the results of this series of experiments show that mixtures of viruses can be used to deliver two transgenes to a target tissue but that this approach is not practicable to achieve high levels of co-expression of two transgenes in individual cells.

**Figure 5:**
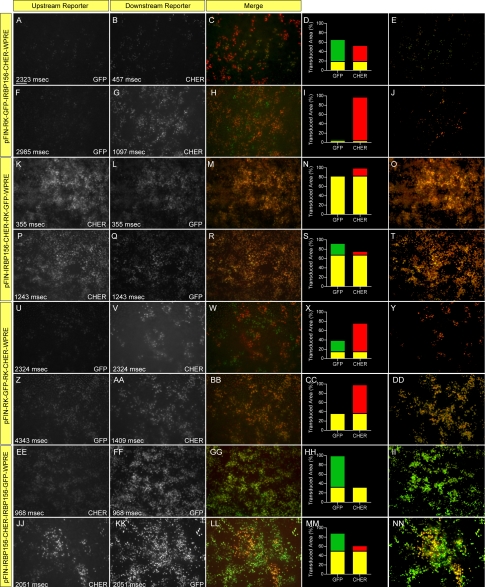
Expression characteristics of dual promoter vectors constructed using rhodopsin kinase (RK) and interphotorecepter binding protein (IRBP)156 promoters. pFIN-RK-GFP-IRBP156-CHER-WPRE (3.3×10^7^ vector genomes/µl; **A-E** and **F-J**), pFIN-IRBP156-CHER-RK-GFP-WPRE (1.5×10^9^ vector genomes/µl; **K-O** and **P-T**), pFIN-RK-GFP-RK-CHER-WPRE (1.85×10^7^ vector genomes/µl; **U-Y** and **Z-DD**), or pFIN-IRBP156-CHER-IRBP156-GFP-WPRE (1.7×10^9^ vector genomes/µl; **EE-II** and **JJ-NN**) lentivirus was injected into the developing neural tubes of E2 chicken embryos in ovo. A minimum of four retinal whole mounts were examined for each virus. Retinal regions shown in the figure were selected to illustrate the range of transgene expression characteristics observed in infected cells. Each region was photographed twice using the exposure duration shown in the lower left of each panel and filters appropriate for detection of CHER or GFP. Each row in the figure shows one selected region. The merged images (**C**, **H**, **M**, **R**, **W**, **BB**, **GG**, **LL**) were analyzed using the co-localization module of the Zeiss AxioVision Image Suite. The results of these analyses are expressed as the percent of the transduced area in the image (pixels) containing co-localized GFP and CHER (yellow bar) or GFP (green bar) or CHER (red bar) fluorescence alone. (**D**, **I**, **N**, **S**, **X**, **CC**, **HH**,** MM).** The images shown in **E**, **J**, **O**, **T**, **Y**, **DD**, **II**, **NN** were extracted from the merged images (**C**-**LL**) and show only those areas of the merged image in which GFP was co-localized with CHER. The scale bar shown in **A** is applicable to all images and equals 50 µm.

**Figure 6:**
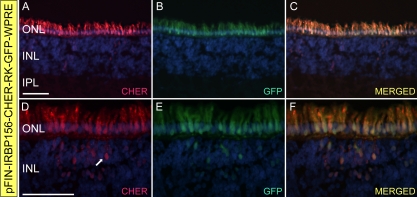
Cellular specificity of the pFIN-IRBP156-CHER-RK-GFP-WPRE dual-promoter vector. pFIN-IRBP156-CHER-RK-GFP-WPRE (1.5×10^9^ vector genomes/µl) lentivirus was injected into the developing neural tubes of E2 chicken embryos in ovo. The retinas of the injected embryos were harvested on E19–20, examined as whole mounts using native fluorescent, and sectioned (10 µm). **A**-**C**: A representative section showing the extent of photoreceptor infection. The merged image indicates that nearly all cells are co-expressing the two fluorescent reporter proteins, CHER and GFP. **D**-**F**: Close up of transduced photoreceptor layer in region containing INL cells expressing the viral transgene (arrow in **D**). All sections were counterstained with DAPI. All scale bars shown equal 50 µm. Abbreviations: ONL represents outer nuclear layer; INL represents inner nuclear layer; IPL represents inner plexiform layer.

Given the results of our mixed virus experiments, we returned our focus to developing dual-promoter vectors that specifically target cones and rods, rods only, or Müller cells and reliably express both transgenes, features that would increase the experimental and potentially the therapeutic usefulness of the vectors. To build the dual promoter vector targeting cones and rods, we selected the IRBP156 (392 bp) and RK (250 bp) promoters, both of which exhibit strong activity in cone and rod cells (Figure 1) and are relatively small in size. We constructed four different dual-promoter reporter vectors using these promoters and examined their expression in vivo: (1) pFIN-RK-GFP-IRBP156-CHER-WPRE; (2) pFIN-IRBP156-CHER-RK-GFP-WPRE; (3) pFIN-RK-GFP-RK-CHER-WPRE; (4) pFIN-IRBP156-CHER-IRBP156-GFP-WPRE (Figure 5). Vectors 1 and 2 were constructed to determine if the order of appearance of the IRBP156- and RK-driven transgenes influenced their expression levels. Vectors 3 and 4 were constructed to determine if photoreceptor-specific dual-promoter vectors carrying two copies of the same promoter can be efficiently packaged into lentivirus and if so, if expression of the two transgenes in vivo is comparable. Comparisons of the expression characteristics of vector 1 - pFIN-RK-GFP-IRBP156-CHER-WPRE (Figure 5A-E,F-J) and vector 2 - pFIN-IRBP156-CHER-RK-GFP-WPRE (Figure 5K-O,P-T) showed that expression of the IRBP156-CHER transgene was higher than that of the RK-GFP transgene regardless of its position in the vector (compare Figure 5B,G to Figure 5A,F and Figure 5K,P to Figure 5L,Q) and was highest when it was located in the upstream position (compare Figure 5K,P to Figure 5B,G). The percent of transduced cells co-expressing detectable levels of GFP and CHER was much greater in retinas transduced with pFIN-IRBP156-CHER-RK-GFP-WPRE (60%–80%; Figure 5N-O,S-T) than in retinas transduced with pFIN-RK-GFP-IRBP156-CHER-WPRE (<20%; Figure 5D-E,I-J). We next examined the performance of vectors 3 - pFIN-RK-GFP-RK-CHER-WPRE and 4 - pFIN-IRBP156-CHER-IRBP156-GFP-WPRE. The estimated titers of each of the viruses generated using these vectors (vector 3, 1.85×10^10^ vector genomes/ml and vector 4, 1.7×10^12^ vector genomes/ml) were comparable to the average titer of all of the viruses that we generated for this study (7.45×10^12^ genomes/ml), indicating that the presence of two identical promoters in the lentivector did not interfere with packaging of the virus. Examination of retinas transduced with either of these vectors revealed that both of the transgenes carried by these vectors were expressed in 10%–50% of the infected cells (Figure 5X,CC,HH,MM). In general, expression of the downstream gene was higher in cells expressing both transgenes (Figure 5; compare Figure 5V,AA to Figure 5U,Z and Figure 5FF,KK to Figure 5EE,JJ). In approximately 35%–70% of the infected areas, only the downstream reporter protein was detected (Figure 5X,CC,HH,MM). In sum, our data indicate that pFIN-IRBP156-CHER-RK-GFP-WPRE is the most efficient vector of the four tested in terms of its ability to consistently express both of the transgenes carried by the vector. As a final test of the utility of this vector, we examined the cellular specificity of the expression of the integrated transgenes in sections of infected retinas. Examination of sections of retinas transduced with pFIN-IRBP156-CHER-RK-GFP-WPRE virus revealed that the individual expression characteristics of these two promoters (Figure 1) were retained in the dual-promoter vector configuration. Robust expression of both CHER and GFP was observed in the photoreceptor cell layer (Figure 6). In addition, a few scattered cells expressing these proteins were also detected in the inner nuclear layer of these retinas (arrow; Figure 6D). These results support use of this dual-promoter vector to target expression of two proteins to cone and rod cells and are summarized in Table 3.

**Table 3:** Summary of dual-promoter vector activities in chicken retina

	**Relative percent of transduced cells expressing reporters**		
**Vector**	**Cells only expressing upstream reporter**	**Cells only expressing downstream reporter**	**Cells expressing both reporters**
RK-GFP-IRPB156-CHER	25	64	11
IRBP156-CHER-RK-GFP	12	13	75
XOPS-tdTOM-MOPS-GFP	35	33	32
MOPS-GFP-XOPS-tdTOM	24	25	51
RK-GFP-RK-CHER	13	62	25
IRBP156-CHER-IRBP156-GFP	6	53	41
RK-GFP-HS4(2x250)F-IRBP156-CHER	3	70	27
RK-GFP-HS4(1.2)F-IRBP156-CHER	25	47	28

**Figure 7:**
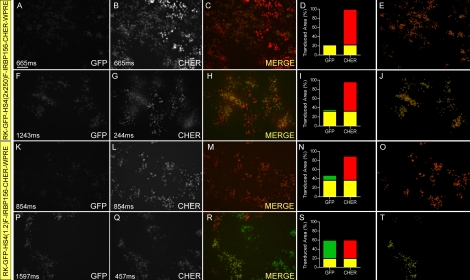
The effects of internal insulators on the expression of the RK-GFP and IRBP156-CHER transgenes carried by the pFIN-RK-GFP-IRBP156-CHER-WPRE vector. Examination of the expression of GFP and CHER in retinas transduced with pFIN-RK-GFP-HS4(2×250)F-IRBP156-CHER-WPRE (3.3×10^8^ vector genomes/µl; **A-J**) or pFIN-RK-GFP-HS4(1.2)F-IRBP156-CHER (1.2×10^8^ vector genomes/µl; **K-T**). Each lentivirus was injected into the developing neural tubes of E2 chicken embryos in ovo. The retinal whole mounts were photographed twice using the exposure duration shown in lower left of each panel and filters appropriate for detection of GFP or CHER. The GFP and CHER images were merged (**C**, **H**, **M**, **R**) and analyzed using the co-localization module of the Zeiss AxioVision Image Suite. The results of these analyses are expressed as the percent of the transduced area in the image (pixels) containing co-localized GFP and CHER (yellow bar) or GFP (green bar) or CHER (red bar) fluorescence alone **D**, **I**, **N**, **S.** The images shown in **E**, **J**, **O**, **T** were derived from the merged images (**C**, **H**, **M**, **R**) and show only those areas of the merged image in which GFP and CHER were co-localized. The scale bar shown in A is applicable to all images and equals 50 µm.

Before leaving our efforts to construct dual-promoter vectors targeting both cones and rods, we conducted a series of experiments to determine if we could improve expression of the upstream transgene RK-GFP in the pFIN-RK-GFP-IRBP156-CHER vector (Figure 5A-E,F-J) by separating the two transgenes carried by the vector with insulator elements, a strategy that has been shown to improve expression levels of two independently regulated transgenes in lentiviral vectors [16,36]. Two vectors were constructed for this experiment by inserting either the core 2×250 bp or the 1.2 kb chicken β-globin HS4 insulator in the forward orientation between the upstream (RK-GFP) and downstream (IRBP156-CHER) transgenes: pFIN-RK-GFP-HS4(2×250)F-IRBP156-CHER-WPRE (Figure 7A-E,F-J) and pFIN-RK-GFP-HS4(1.2)F-IRBP156-CHER-WPRE (Figure 7K-O,P-T). Examination of retinas transduced with these vectors showed that insertion of either insulator between the transgenes did not markedly increase expression levels of the upstream GFP reporter protein relative to the downstream CHER reporter protein (compare Figure 7A to Figure 7B and Figure 7K to Figure 7L). There was some evidence that the relative percent of the transduced cells that expressed both reporter proteins was slightly higher in the presence of the insulators, but clearly the expression levels of both proteins did not approach those obtained from the pFIN-IRBP156-CHER-RK-GFP-WPRE vector. The maximum percent of the transduced area of retinas expressing both transgenes with the non-insulated vector was about 19% (Figure 5D), whereas the maximum percents for retinas transduced with pFIN-RK-GFP-HS4(2×250)F-IRBP156-CHER-WPRE or pFIN-RK-GFP-HS4(1.2)F-IRBP156-CHER-WPRE were 32% (Figure 7I) or 36% (Figure 7N), respectively. Based on these analyses, we concluded that the slight improvement that we observed in transgene co-expression in transduced cells, while of interest, was not sufficient to continue to pursue further development of these vectors (results summarized in Table 3).

**Figure 8:**
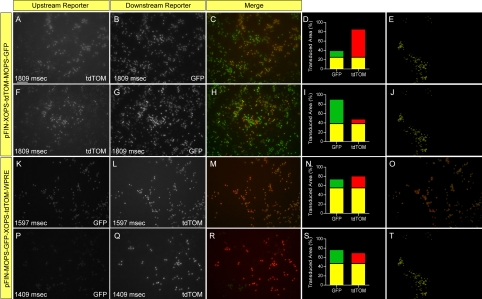
Expression characteristics of dual promoter vectors constructed using murine opsin promoter (MOPS) and *Xenopus* opsin promoter (XOPS) promoters. pFIN-XOPS-tdTOM-MOPS-GFP (1.6×10^8^ vector genomes/µl; **A-J**) or pFIN-MOPS-GFP-XOPS-tdTOM-WPRE (4.4×10^8^ vector genomes/µl; **K-T**) lentivirus was injected into the developing neural tubes of E2 chicken embryos in ovo. Retinal whole mounts (one retina per horizontal row) were photographed twice using the exposure duration shown in lower left of each panel and filters appropriate for detection of tdTOM or GFP. The merged images (**C**, **H**, **M**, **R**) were analyzed using the co-localization module of the Zeiss AxioVision Image Suite. The relative percent area (pixels) of each image containing GFP (green bar) or tdTOM (red bar) fluorescence alone or both GFP and tdTOM (yellow bar) is shown in panels **D**, **I**, **N**, **S.** The images shown in **E**, **J**, **O**, **T** were derived from the merged images (**C, H, M, R**) and show only those areas of the merged image in which GFP was co-localized with CHER. The scale bar shown in **A** is applicable to all images and equals 50 µm.

Next, we focused on building a dual promoter vector that targets rod cells and reliably expresses both of the transgenes carried by the vector. To build this vector we used the XOPS (1,370 bp; Figure 1) and MOPS (477 bp [18];) promoters, both of which exhibit strong specific activity in rod cells. We constructed two different dual-promoter reporter vectors using these promoters and examined their expression in vivo: pFIN-XOPS-tdTOM-MOPS-GFP and pFIN-MOPS-GFP-XOPS-tdTOM-WPRE. The fluorescent signals generated by tdTOM and GFP in retinas infected with pFIN-XOPS-tdTOM-MOPS-GFP were easily detected, but the levels of expression of each protein varied within transduced areas with less than 50% of the transduced cells expressing enough of both of these proteins to be identified as tdTOM and GFP positive (Figure 8A-E: 25% co-localized, F-J: 38% co-localized). Neither transgene was preferentially expressed in transduced cells. In some areas (Figure 8A-E), only expression of the upstream transgene (tdTOM) was detected (60% of the cells in the transduced region), whereas in other areas (Figure 8F-J), only expression of the downstream transgene (GFP) was detected (52% of the cells in the transduced region). Reversing the positions of the XOPS and MOPS promoter-driven transgenes in the vector altered the expression profile of the two transgenes. In retinas infected with pFIN-MOPS-GFP-XOPS-tdTOM-WPRE, the fluorescent signal generated by the upstream reporter (GFP) could be detected in transduced regions but the signal was faint compared to that generated by the downstream reporter (tdTOM; Figure 8 compare Figure 8K,P to Figure 8L,Q). Even though the GFP signal was faint, it was detectable in many of the transduced cells. Unlike the results obtained using pFIN-XOPS-tdTOM-MOPS-GFP, approximately 50% of the transduced cells expressed enough of both reporter proteins to be identified as GFP and tdTOM positive (Figure 8K-O: 55% co-localized, P-T: 47% co-localized). Taken together, the results obtained from these two dual promoter vectors do not point to a “most optimal” vector; however, if co-expression of both transgenes in infected cells is the overarching goal, then pFIN-MOPS-GFP-XOPS-tdTOM-WPRE would seem to be the vector of choice (see Table 3 for result summary).

**Figure 9:**
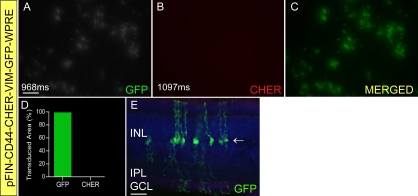
Expression characteristics of a dual promoter vector constructed using cluster differentiation (CD)44 and vimentin (VIM) promoters. pFIN-CD44-CHER-VIM-GFP-WPRE lentivirus was injected into the developing neural tubes of E2 chicken embryos *in ovo*. Retinal whole mounts were photographed twice using filters appropriate for detection of GFP (**A**) or CHER (**B**) and the exposure times (ms) shown in the lower left of the images. The GFP and CHER images were merged (**C**) and analyzed using the co-localization module of the Zeiss AxioVision Image Suite. The relative percent area (pixels) of the merged image containing GFP (green bar) or CHER (red bar) fluorescence alone or both GFP and CHER (yellow bar) is shown in panel **D.** Sections of the transduced retinas revealed that GFP expression was largely restricted to Müller cells (arrow, **E**). A few GFP-positive horizontal cells were detected in these retinas but their numbers were reduced relative to the numbers of these cells that were present in retinas transduced with pFM-VIM-GW (Figure 2J-N). Scale bars shown in **A** and **E** equal 50 µm.

Finally, we built and tested one dual-promoter vector to target Müller cells. Our analyses of the VIM, CD44 and GFAP promoters showed that the GFAP promoter was the only promoter that was expressed exclusively in Müller cells (Figure 2). Although we found that the GFAP promoter possessed the desired specificity, we also found that the levels of expression of this promoter in retinas unaffected by injury or disease were not high enough to produce detectable amounts of reporter protein. Even though the CD44 and VIM promoters did not exhibit the cellular specificity shown by the GFAP promoter, we chose to pair them in building the pFIN-CD44-CHER-VIM-GFP-WPRE vector. Our decision to pair these promoters was prompted, in part, by our previous observation that the specificity of two promoters can be enhanced when paired in the dual-promoter configuration [18]. Examination of retinas transduced with pFIN-CD44-CHER-VIM-GFP-WPRE revealed a somewhat unexpected result (Figure 9). Only the downstream reporter protein (GFP) was detected using fluorescent microscopy (Figure 9A,C,D). The levels of the upstream reporter protein (CHER), if produced, were not high enough to permit direct visualization of the native protein (Figure 9B,D). Interestingly, the activity of the VIM promoter was more restricted so that only a few GFP-positive horizontal cells were detected (Figure 9E) relative to the numbers of these cells observed in retinas transduced with pFIM-VIM-GW (Figure 2). Clearly, this dual-promoter vector did not meet our performance criteria for generating detectable amounts of the fluorescent reporter proteins encoded by the two transgenes carried by the vector.

## Discussion

Viral vectors are useful vehicles for delivering genes to populations of cells. In many cases, it is desirable to limit expression of these genes to specific subpopulations of cells. For many retinal diseases, photoreceptor cells are the targeted cell subpopulation. We have now completed in vivo analyses of seven promoters in chicken retina and have identified two promoters that are active in cone cells (GCAP292 and IRBP1783) [18], three promoter that are active in cone and rod cells (IRBP156, RK, NRLL; Figure 1), and two promoters that are active in rod cells (MOPS [18] and XOPS; Figure 1). Of these seven, GCAP292 (601 bp), IRBP156 (350 bp), and RK (292 bp) also exhibited minor activity in cells located in the inner nuclear layer. Since one of the goals of this study was to develop dual-promoter lentiviral vectors targeting photoreceptors, we deemed that the slight loss in cellular specificity exhibited by these promoters would be acceptable in view of the relatively small size of these promoters that is advantageous when constructing lentiviral vectors.

In addition to our analyses of photoreceptor promoters, we also examined the activities of three putative glial-specific promoters, VIM, CD44 and GFAP, with the aim of developing a dual-promoter lentiviral vector targeting Müller cells. These promoters have been reported to be glial-specific in rat retina when incorporated into lentiviral vectors pseudotyped with vesicular stomatitis virus glycoprotein and delivered via subretinal injection [25]. The results of our analyses showed that the activities of the CD44 and VIM promoters in chicken retina were not limited to Müller glia. CD44-driven GFP was detected in all retinal cell types including Müller cells, and VIM-driven GFP was detected primarily in horizontal and Müller cells (Figure 2). The only promoter that exhibited Müller cell-specific activity in chicken retina was the GFAP promoter and its level of activity was positively correlated with the presence of retinal injury or disease, an activity profile that matches that observed in the S334Ter^+^/^−^ transgenic rat [25]. Our observation that the CD44 and VIM promoters are active in multiple cell types in chicken retina was unexpected in view of previous analyses of the activities of these promoters in rat retina that showed that the activities of these promoters were restricted to Müller cells [25]. While we do not know the reason for the differences observed in the cellular specificities of these promoters in chicken and rat retina, it is unlikely that they reflect differences in the lentiviral vectors used in these studies because (1) the VIM lentivirus was packaged using the same vector that was used in the rat study and (2) the CD44 promoter used in our study was removed from the pFmCD44.1GW lentivector used in the rat study [25] and placed into our pFIN lentivector backbone [18] which is very similar to the pFUGW lentivector backbone [37] that was used to create pFmCD44.1GW. We propose that the differences observed in the cellular specificities of these promoters reflect differences in the timing and routes of delivery of the viruses used in these studies. In the rat study, the viruses were injected into the subretinal space at postnatal days 1 and 21; whereas in our study, the viruses were injected into the neural tubes of E2 chicken embryos when the optic vesicles have formed and the neural tube is closing. The biggest difference between these approaches is the types of cells that come into contact with the injected lentiviruses. The subretinal approach, while maximizing exposure of the retinal pigment epithelium, photoreceptors and Müller cells to virus, limits exposure of the cells of the inner retina to virus. Delivery of virus to the developing embryo, on the other hand, exposes retinal progenitor cells to virus whose daughter cells, each of which carry the integrated viral transgene, differentiate to form the entire neural retina. The observations that AAV2-CD44-GFP was active in a subset of retinal ganglion cells [25] and that GFP was present in amacrine and ganglion cells in CD44-GFP transgenic mice [38] are consistent with our observation that the CD44 promoter is capable of activity in a broader array of cell types than has been previously suggested by studies of mouse retina showing that the CD44 protein is restricted to Müller cells [39]. Together, these observations serve to highlight the importance of considering developmental stage, viral type, and delivery route when characterizing the expression characteristics of promoters in vivo.

Much of our effort in this study was directed toward developing dual-promoter vectors that specifically target cones and rods, rods alone, and Müller cells. For the vector targeting cones and rods, the performance of the dual promoter vector carrying the IRBP156 and RK promoters with IRBP156 in the upstream position was the most consistent of those tested, exhibiting high levels (~70%) of expression of both of the proteins carried by the vector in transduced cone and rod cells. Reversal of the order of the appearance of the two promoters in the dual promoter vector dramatically reduced the expression levels of the upstream gene driven by RK, a phenomenon termed promoter suppression [40]. Unequal expression of one of the cistrons carried by bicistronic constructs is often problematic when developing lentiviral vectors carrying multiple, heterologous promoters [40,41]. We do not know the mechanism underlying the interference exhibited by this vector, but since it is dependent on promoter order and the levels of expression of the upstream gene are compromised, it may be that transcription of the downstream gene induces structural changes in the bicistronic transgene that prevents efficient transcription from the upstream promoter. To see if we could ameliorate suppression of expression of the upstream cistron, we inserted an HS4 insulator between the RK-GFP and IRBP156-CHER cistrons. This approach has been successfully used by others to overcome positional effects that adversely impact the expression of individual cistrons carried by multi-gene lentiviral vectors [36]. We found that the relative level of expression of the RK-GFP cistron to that of the IRBP156-CHER cistron following insertion of either the 2×250 or the 1.2-kb HS4 insulator between the cistrons improved slightly but did not approach that obtained from the vector carrying the cistrons in the reverse order (pFIN-IRBP156-CHER-RK-GFP-WPRE). Further improvements in the performance of this vector could potentially be achieved by cloning the cistrons into the vector backbone in antisense orientation and adding polyadenylation signals to each cistron [16] or by inserting a second WPRE element between the two cistrons [42].

In addition to the dual promoter photoreceptor-specific vectors that we constructed, we also constructed and tested the performance of a dual promoter vector that was designed to target Müller cells. In designing this vector, we paired the CD44 and VIM promoters to create pFIN-CD44-CHER-VIM-GFP-WPRE. Both of these promoters are active in Müller cells but are also active in non-glial cells. We hypothesized, based on our previous observation that pairing promoters can increase the specificity of each promoter [18], that placing these two promoters in a tandem *cis* arrangement would enhance the glial specificity of the other. The result obtained using this vector was somewhat unexpected. Expression of CHER from the upstream CD44-CHER cistron was undetectable, while expression of GFP from the downstream VIM-GFP cistron was detected primarily in Müller cells with little GFP detected in horizontal cells, an observation that suggests that pairing VIM with CD44 increased the specificity of the VIM promoter with regard to glial cells. Our goal was to obtain a vector that efficiently drives expression of two proteins in transduced Müller cells. Given the results obtained with this vector, we may be able to accomplish this goal by either developing a dual promoter vector in which we pair the GFAP promoter with the VIM promoter or by developing a multi-cistron transgene driven by either the GFAP or VIM promoter that contains self-cleaving 2A-like peptides that promote generation of multiple proteins from a single transcript [25].

Our goal for this study was to develop dual-promoter lentiviral vectors that exhibited photoreceptor or Müller cell-specific expression that consistently produced two proteins in the targeted cells. Using combinations of photoreceptor promoters we were able to develop vectors that are expressed in specific populations of retinal cells, namely vectors that target cone and rod cells (pFIN-IRBP156-CHER-RK-GFP-WPRE) or that target rod cells only (pFIN-MOPS-GFP-XOPS-tdTOM-WPRE). Analyses of the expression of the two proteins encoded by these vectors using direct fluorescence revealed that detection of one protein was predictive of the presence of the second protein in greater than 50% of the infected cells. For the pFIN-IRBP156-CHER-RK-GFP-WPRE vector this percentage was closer to 80%, a value that would likely have been higher if antibodies had been used to aid in the visualization of the fluorescent proteins. These vectors should be useful in studies of retina when co-delivery of a reporter protein with an experimental protein is desired or when expression of two exogenous proteins in targeted cells is required.
